# Theory-based educational intervention on oral hygiene behavior among university students: a randomized controlled trial

**DOI:** 10.1038/s41405-025-00368-y

**Published:** 2025-09-26

**Authors:** Rieski Prihastuti, Daisuke Hinode, Makoto Fukui, Omar M. M. Rodis, Yoshizo Matsuka

**Affiliations:** 1 Department of Stomatognathic Function and Occlusal Reconstruction, Graduate School of Biomedical Sciences, Tokushima University, Tokushima, Japan; 2 Dental Hygiene Program, Faculty of Dentistry, Universitas Gadjah Mada, Yogyakarta, Indonesia; 3 Department of Hygiene and Oral Health Science, Graduate School of Biomedical Sciences, Tokushima University, Tokushima, Japan; 4 Department of International Oral Health Science Education, Graduate School of Biomedical Sciences, Tokushima University, Tokushima, Japan

**Keywords:** Oral hygiene, Dental epidemiology

## Abstract

**Objective:**

The objective was to assess the effectiveness of the theory-based educational intervention tailored to the Theory of Planned Behavior (TPB) and behavior change techniques (BCTs) on oral hygiene behavior among university students through the oral hygiene behavior index and oral health outcomes.

**Materials and methods:**

A randomized controlled trial involving university students was conducted from November 2022 to March 2023 (*n *= 71). Participants were randomly assigned to a control group (*n *= 38) or an intervention group (*n *= 33). The control group received conventional dental health education, whereas the intervention group underwent a 21-day TPB-based educational intervention delivered through a three-minute video. Oral hygiene behavior index (OHBI) and oral health outcomes, including caries activity, salivary hemoglobin level, and total bacterial count, were assessed at baseline and three-month post-intervention.

**Results:**

Statistically significant improvements were seen in the OHBI score between groups and within the intervention group at three-month follow-up, with higher scores indicating better oral hygiene behavior. In addition, caries activity and salivary hemoglobin level were also improved. The total bacterial count in the intervention group did not show any statistically significant difference, even though it was lower than that of the control group.

**Conclusion:**

This study indicates that theory-based educational intervention tailored to TPB and BCTs improves oral hygiene behavior and certain oral health outcomes. Future studies should examine the long-term effects and generalizability in diverse populations.

**Trial registration:**

Thailand Clinical Trial TCTR20230105003.

## Introduction

Untreated oral health problems, mainly dental caries and periodontal disease, experienced by nearly 3.5 million people not only cause tooth loss but also have a negative influence on day-to-day functioning and result in a lower quality of life [1, 2]. Data from the Indonesia Basic Health Research [3] showed that 57.6% of residents had oral health problems, and only 10.2% had been treated, including young adults. The 2023 Indonesian Health Survey highlighted that young adults aged 15–24 had the highest prevalence of bleeding gums (8.7%) among all age groups. While 72.8% of young adults brush their teeth twice daily, only 7.4% brush their teeth in the morning after breakfast and before bed, indicating poor behavioral compliance and a knowledge behavior gap [4]. Behavioral interventions grounded in psychological theory are essential to address the gap. The theory of planned behavior (TPB) has been frequently used to effectively explain health-related and non-health-related behaviors such as smoking, dietary, oral health, and oral hygiene [5–9]. According to TPB, intention is the factor that influences someone to adopt a behavior that promotes their health, and is affected by attitude, social norm, and perceived behavioral control, with attitude as the strongest predictor [10]. Therefore, an effective intervention should focus on attitudinal change. Behavior change techniques (BCTs), such as providing information about health impacts and guidance on performing the behavior, correlated not only with knowledge, skills, beliefs about consequences, but also attitude toward behavior, perceived susceptibility/vulnerability, and intention [11]. These BCTs were most used in behavioral interventions [12]. Furthermore, intervention effectiveness is also influenced by the mode of delivery [13].

Several experimental studies have reported the effectiveness of various interventions in enhancing oral hygiene behavior in a targeted population. However, few research studies have targeted young adults [14]. Despite oral health knowledge and oral hygiene behavior, intervention studies often include clinical outcome measures. However, most rely heavily on labor-intensive and time-consuming measures (bleeding on probing, plaque scores, pocket depth, and clinical attachment loss), which are impractical for a community setting [15–18]. Our study addresses this gap by employing rapid, non-invasive alternatives, namely the caries activity test, salivary hemoglobin level, and total bacterial count.

This study evaluates the effect of a theory-based educational intervention based on TPB and BCTs on oral hygiene behavior among university students. We hypothesized that the intervention group would demonstrate better oral hygiene behavior and improved oral health outcomes compared to controls.

## Materials and methods

### Study design and inclusion criteria

This study was a 1:1 allocation randomized controlled parallel trial. A computer-generated list from www.randomization.com was used to perform participant randomization. Data were inputted and randomized by a dental hygienist according to the saliva examination order. Similarly, a single-blind, oral examiner-blind design was adopted in this study. University students from non-medical fields between 18 and 25 years old were eligible subjects. Students who had DMF-T ≥ 7, had fixed orthodontic or prosthodontic appliances, were on antibiotics, or had used chlorhexidine mouthwash within two weeks before the recruiting date were excluded from participating. The subjects were recruited from The Faculty of Teacher Training and Education, Ahmad Dahlan University, Indonesia. All procedures adhered to the Declaration of Helsinki. This study was approved by the Ethics Committee Faculty of Dentistry, Universitas Gadjah Mada (217/UNI/KEP/FKG-RSGM/EC/2022) and registered in the Thai Clinical Trial Registry (TCTR20230105003), which can be accessed at https://www.thaiclinicaltrials.org/. Participants provided written informed consent prior to enrollment. In addition, this study followed the CONSORT statement.

### Data collection

All participants completed the preliminary screening questionnaire, and those who satisfied the inclusion criteria then underwent an oral examination to establish the status of the caries experience. Following that, those with DMF-T < 7 underwent salivary examinations, including caries activity test, saliva hemoglobin level, and total bacterial count. They were also asked to complete several questionnaires, including a questionnaire on oral health knowledge (OHK) and the oral hygiene behavior index (OHBI) [8, 19]. The DMFT scoring was performed by calibrated examiners. The intraclass correlation coefficient was 0.75, indicating good agreement between examiners. Caries activity test and salivary hemoglobin level were rated by two examiners who reached consensus based on visual color interpretation, while total bacterial counts were determined by the device automatically.

### Intervention

Maintaining good oral hygiene behavior involves several key points, such as brushing two times a day (after breakfast and before bedtime) using a soft-bristled toothbrush and fluoride toothpaste with minimal pressure for two minutes, and practicing daily interdental cleaning and tongue cleaning to remove plaque and bacteria [19]. To reinforce these, an oral health intervention was designed in a 21-day video-based program via web platforms. The video was intentionally designed to last for three minutes. Toothbrushing alone is generally recommended for 2 min, with additional time allocated for interdental and tongue cleaning. By coordinating the duration of the video with the task, the intervention aimed at strengthening behavior and enabling students to incorporate learning materials into their daily lives. While the video was intended to be watched during toothbrushing, participants were also able to view it at other times. Furthermore, the intervention was conducted for 21 days, which aligned with the previous studies [15, 20]. The educational material has been adopted and modified to the OHK and OHBI, which were developed based on the TPB construct. Several BCTs, such as providing information on consequences and giving oral hygiene instructions, were also implemented in the intervention [8, 19]. During the first week, videos about the oral cavity and oral health problems were introduced as established foundational knowledge. In the second week, videos describing oral health problems related to nutrition and smoking and their impact on general health were then explained. In the last week, plaque management, comprising toothbrushing, interdental brushes, and tongue cleaning, was introduced as an essential strategy to maintain oral hygiene. A video was followed by a simple quiz to reinforce learning, and summary videos were presented on days 7, 14, and 21 to review the materials from the preceding week and enhance knowledge retention. The effectiveness of this educational intervention will be assessed through an OHK and OHBI questionnaire administered three months after its completion. Monitoring was conducted using the video completion tracking feature integrated into the web-based platform. Incomplete viewers were excluded to ensure data reliability. On the other hand, the control group received conventional dental health education. At the start of the study, all participants, including the control group, were given brief oral health instructions covering toothbrushing techniques, recommended duration, and frequency. These instructions were provided immediately after the dental examination to ensure ethical treatment of all participants. After randomization, the control group did not receive any intervention.

### Outcomes

The outcome of the study was oral hygiene behavior change, which was assessed through the OHBI questionnaire. This questionnaire was developed based on the TPB [19]. This questionnaire explores several oral hygiene items, including toothbrushing frequency, toothbrushing moments, toothbrushing force, toothbrushing duration, toothbrushing method, fluoride toothpaste, interdental cleaning, and tongue cleaning. The OHBI score goes from 0 to 17, with a higher score suggesting good oral hygiene behavior. In addition, the OHK questionnaire comprises 11 true/false questions, with a higher score indicating better OHK. The OHBI and OHK questionnaires showed high internal consistency (α = 0.65 and α = 0.74, respectively) and correlated significantly with TPB constructs [8]. The Indonesian version of OHBI showed good validity and reliability when implemented in adolescents [21]. To further evaluate its reliability in university students, we conducted a test-retest analysis. The results indicated that the OHBI questionnaires had good reliability (α = 0.87).

Oral health status, determined by the caries activity test, salivary hemoglobin level, and total bacterial count, was also assessed. These outcomes were objective measurements that served as complementary validation of the effect of behavior change. Food, drink, and oral hygiene were prohibited for all participants for at least two hours before the oral assessment. Saliva was collected around 10 and noon to reduce the impact of diurnal fluctuations on the components of saliva.

#### Caries activity test

Caries activity test was measured using the resazurin disc test (GC Showa, Japan) for evaluating the cariogenic bacterial activity. A 0.03 mL saliva sample from each participant was placed at the center of a disc of paper. The disc was then incubated at a temperature of 32–37 °C for 15 min to activate cariogenic bacteria. Instead of using an incubator, which requires a long time, the disc can be placed on the upper arm with the sleeve down to maintain optimal temperature [22]. Results were indicated by three color gradations, indicating the degree of caries activity from the interaction between bacteria and saliva. Maki et al. [23] demonstrated that resazurin discs were highly sensitive to gram-positive microorganisms such as *S. mutans, S.mitis, S. faecalis, S. aureus, L. casei*, and *B. subtilis*. The test’s sensitivity also correlated with the number of *S. mutans* and *Lactobacilli* present in saliva. While this test is commonly used in Japan to monitor oral hygiene, evidence is limited, and recent research has reported an association between elevated resazurin scores with poorer oral health status was based on very small samples [24].

#### Salivary hemoglobin level

Salivary hemoglobin levels were measured using Perioscreen (Sunstar, Osaka, Japan). Approximately 1 mL of saliva was collected and diluted five times with water to prepare the sample for measurement. The Perioscreen strip is then immersed in the saliva sample. After 5 min, the color change in the strip was compared with the manufacturer’s to estimate the hemoglobin level, which was categorized into 0, 2, and 5 μg/mL. Previous studies confirmed its high sensitivity in correlating with the percentage of BOP and pocket depth <4 mm [25]. Compared to the salivary multi-test, Perioscreen showed more sensitivity in assessing periodontal conditions. Additionally, it can be used to guide, assess, and motivate patients [26].

#### Total bacterial count

A dorsum tongue swab was collected and analyzed with a fast bacterial quantification technique (Panasonic Healthcare Co. Ltd., Tokyo, Japan). The bacteria were quantified using a dielectrophoretic impedance measuring (DEPIM) system. Total bacterial counts measured utilizing DEPIM correlated with standard plate counting and mixed bacterial suspension [27, 28]. Furthermore, total bacterial count was found to be associated with oral hygiene, with a higher bacterial count indicating poor oral hygiene [29]. In our study, the bacterial count was used solely to quantify changes in total bacterial load before and after intervention.

### Sample size

A pilot study involving 27 students was carried out before this study. An effect size of 0.74 resulting from the pilot study was used to estimate the sample size. The calculation was carried out using the most recent G*Power (version 3.1.9.6) on a two-tailed t-test [30, 31]. The minimum sample size of 31 participants for each group was necessary, with a power level of 0.80 and a significant difference threshold of 0.05. The number of participants in each group was increased to 40 to allow for potential loss to follow-up throughout 3 months.

### Statistical analysis

The data was analyzed utilizing STATA version 19. Normality of continuous variables was assessed using the Shapiro–Wilk test. Baseline differences between intervention and control groups were evaluated using independent *t*-tests or Mann–Whitney U tests for continuous variables, and chi-square or Fisher’s exact tests for categorical variables. To examine attrition bias, completers and dropouts were compared at baseline using the same approach. Within-group changes from baseline to follow-up were analyzed using paired *t*-tests or Wilcoxon signed-rank tests for continuous variables, and the marginal homogeneity test for categorical variables. Statistical significance was set at *p* < 0.05, and effect size for the OHBI was estimated using Cohen’s d.

## Results

**Fig. 1:**
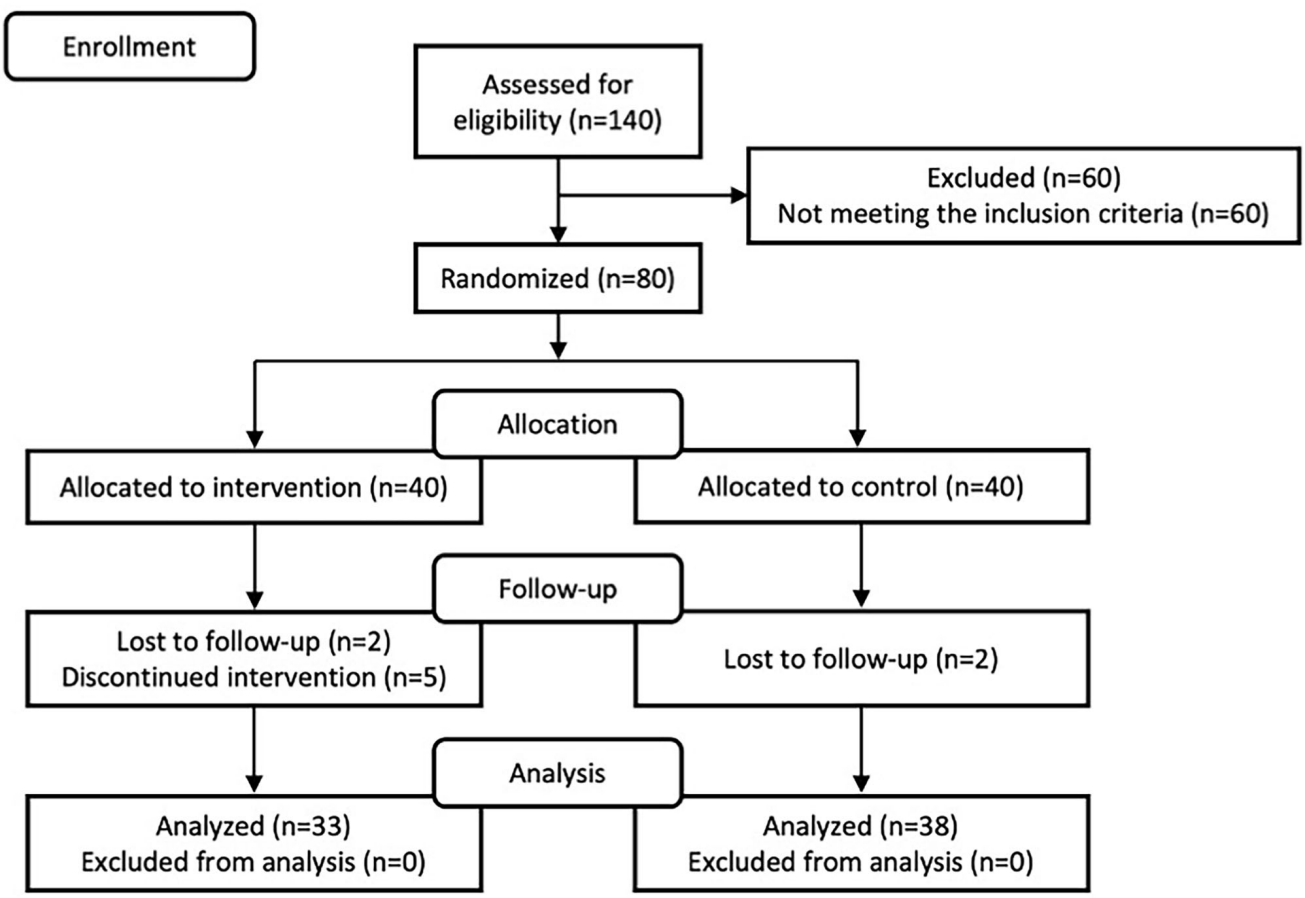
CONSORT flow diagram of the participants. The diagram illustrates the number of participants at each phase with arrows indicating the flow.

Between November and December 2022, 140 students were screened, and 60 of them were found to be ineligible. Eighty students have been randomly assigned to the control and intervention groups. However, only 71 students were included in the final analysis, as shown in Fig. 1. Attrition rates were 17.5% in the intervention group and 5.0% in the control group. The difference was not statistically significant (Fisher’s exact test, *p* = 0.098). Additionally, baseline characteristics of completers and dropouts showed no statistically significant differences (*P* value > 0.05), as shown in Supplementary Table 1. The majority of them were female, and no difference was found in the mean DMF-T score between the control and intervention groups (3.61 ± 2.25 and 3.82 ± 2.24, respectively; *P* value = 0.69). Additional demographic and clinical variables are shown in Table 1. Prior to the intervention, except for the median ages of the students and the caries activity test, the findings demonstrated no significant difference between groups.

**Table 1:** Demographic and oral health outcomes of the participants at baseline.

Characteristics	Control group *n *= 38	Intervention group *n *= 33	*P* value
Gender, *n* (%)			
Female	29 (76.3)	23 (69.7)	0.530^a^
Male	9 (23.7)	10 (30.3)	
Age (years), median (IQR)	18.5 (18, 20)	20 (20, 21)	0.007^b^
Family income, *n* (%)			
Low	6 (15.8)	5 (15.2)	0.972^a^
Moderate	22 (57.9)	20 (60.6)	
High	10 (26.3)	8 (24.2)	
Mother education, *n* (%)			
Low	5 (13.2)	5 (15.2)	0.641^a^
Moderate	18 (47.4)	13 (39.4)	
High	15 (39.5)	15 (45.5)	
Father education, *n* (%)			
Low	5 (13.2)	4 (12.1)	0.878^a^
Moderate	18 (47.4)	14 (42.4)	
High	15 (38)	15 (45.5)	
Oral hygiene instruction, *n* (%)			
No	12 (31.6)	12 (36.4)	0.671^a^
Yes	26 (68.4)	21 (63.6)	
Dental visit, *n* (%)			
No	33 (86.8)	31 (93.9)	0.438^c^
Yes	5 (13.2)	2 (6.1)	
Smoking status, *n* (%)			
Current smoker	4 (10.5)	3 (9.1)	1.000^c^
Former smoker	3 (7.9)	2 (6.1)	
Non-smoker	31 (81.6)	28 (84.8)	
DMF-T	3.61 ± 2.25	3.82 ± 2.24	0.692^d^
OHK	8.55 ± 1.33	8.27 ± 1.55	0.415^d^
OHBI	10.45 ± 2.42	9.76 ± 2.06	0.205^d^
Caries activity test, *n* (%)			
Low	12 (31.6)	10 (30.3)	0.048^a^
Middle	22 (57.9)	12 (36.4)	
High	4 (10.5)	11 (33.3)	
Salivary haemoglobin, *n* (%)			
Negative	8 (21.1)	11 (33.3)	0.517^a^
Positive (2 μg/mL)	10 (26.3)	7 (21.2)	
Positive (5 μg/mL)	20 (52.6)	15 (45.5)	
Total bacterial count (Log10 CFU/mL), median (IQR)	6.29 (6.00, 6.55)	6.20 (5.91, 6.59)	0.836^b^

*IQR* Interquartile range, *DMF-T* decayed, missing, and filled teeth, *OHK* oral health knowledge, *OHBI* oral hygiene behavior index, *CFU* colony forming unit.

^a^Chi-square test.

^b^Mann–Whitney test.

^c^Fisher’s exact test.

^d^Independent *t*-test.

### Oral hygiene behavior index

The OHBI score for the baseline was 9.76 ± 2.06 and 10.45 ± 2.42 in the intervention and control group, respectively. This value increased to 10.79 ± 1.96 in the intervention group, whereas it decreased to 9.76 ± 1.87 in the control group. A statistically significant within-group difference was observed in the intervention group (*P* value = 0.042, Cohen’s d = 0.368), and the between-group difference at follow-up was also significant (*P* value = 0.028, Cohen’s d = 0.595), as shown in Table 2. Furthermore, the difference in delta values between groups was also significant (*P* value = 0.015). Similarly, for OHK, the intervention group improved from 8.27 ± 1.55 at baseline to 8.94 ± 1.22 at follow-up (*P* value = 0.046, d = 0.386). The between-group difference in OHK at follow-up was significant (*P* value = 0.032, d = 0.420), indicating a small-to-moderate positive effect of the intervention on oral health knowledge.

**Table 2:** Oral health knowledge and oral hygiene behavior index of the participants.

Variable	Group	Baseline	Follow-up	Δ	*P* value	Cohen’s d	
						Within group	Between group
OHK	Intervention	8.27 ± 1.55	8.94 ± 1.22	0.67 ± 1.73	0.046^a^	0.386	
	Control	8.55 ± 1.33	8.16 ± 1.70	−0.08 ± 1.82	0.208^a^	−0.043	
	*P* value	0.415^b^	0.032^b^	0.082^b^			0.420
OHBI	Intervention	9.76 ± 2.06	10.79 ± 1.96	1.03 ± 2.80	0.042^a^	0.368	
	Control	10.45 ± 2.42	9.76 ± 1.87	−0.68 ± 2.94	0.182^a^	−0.233	
	*P* value	0.205^b^	0.028^b^	0.015^b^			0.595

Baseline, follow-up, and Δ values are presented as mean ± SD.

Δ change between baseline and follow-up, *OHK* oral health knowledge, *OHBI* oral hygiene behavior index.

^a^Paired *t*-test.

^b^Independent *t*-test.

### Oral health outcomes

**Fig. 2:**
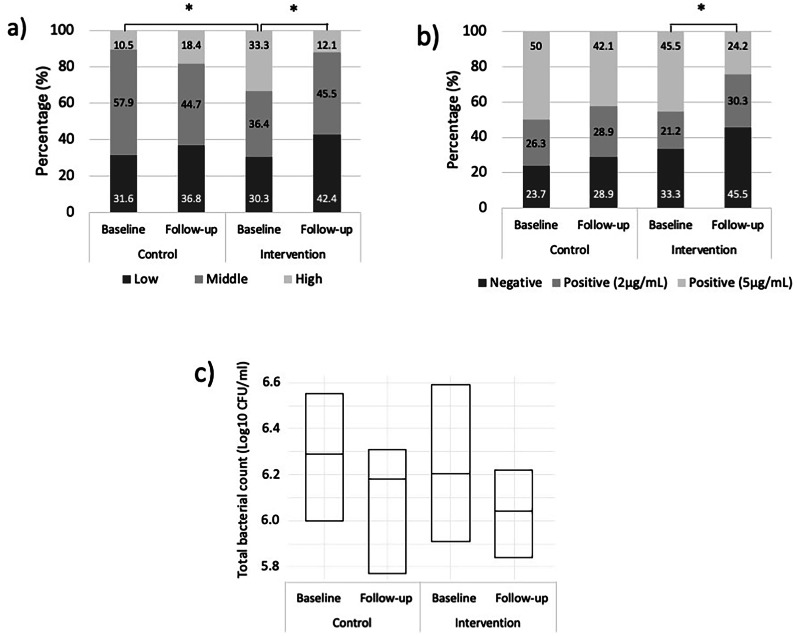
Oral health outcomes change. **a** Caries activity test; **b** salivary hemoglobin level; and **c** total bacterial count. Panels (**a**) and (**b**) are presented as percentages, while (**c**) is presented as median and interquartile range. Asterisks indicate statistically significant differences (*p* value < 0.05). Comparisons without asterisks were not statistically significant.

In Fig. 2, there was a statistically significant difference between groups at baseline in the caries activity test. A statistically significant improvement was also observed within the intervention group. It was also revealed from the marginal homogeneity test for the salivary hemoglobin level that there was a significant difference within the intervention group. Additionally, the total bacterial count in both groups showed a decreasing trend. However, the paired *t*-test revealed no difference between groups at the baseline and 3-month follow-up in the intervention group (*P* value > 0.05).

## Discussion

We evaluated the intervention effects using the OHBI questionnaires, developed based on TPB constructs. Our findings demonstrate that TPB-based interventions have significantly improved the outcomes of OHK, OHBI, and oral health outcomes, such as caries activity test and salivary hemoglobin level among Indonesian university students. The increased OHBI suggests that participants have translated knowledge into action, probably attributed to improved consciousness of the significance of oral health and motivation to adopt healthier behavior. These results are consistent with studies showing that interventions based on TPB improve oral self-care. Raheleh Soltani et al. [32] reported an improvement in the frequency of toothbrushing in children, while Mohammadkhah et al. [33] reported an improvement in oral health behavior in pregnant women. In particular, Darwita et al. [15] demonstrated that a 21-day web application intervention significantly improved dental students’ knowledge and motivation. This result was similar to our results, where the web-based platform probably contributed to an improvement in the OHBI score. Video use may make educational material easier to understand, and the web-based platform media allows students to access the material from anywhere at any time, and it can be repeated [15, 34, 35]. Despite this finding, the control group demonstrated a decreased OHBI score, suggesting a worsening oral hygiene behavior. Evidence showed that repetition in educational intervention is key to improving oral health practices [36]. Without repeated educational intervention, oral hygiene behaviors can deteriorate over time. The intervention group, on the other hand, demonstrated improved behaviors, highlighting the need for repetition.

Findings on oral health outcomes varied across different indicators. First, even though an improvement in caries activity was observed in the intervention group, the results should be interpreted with caution. Despite randomization, a significant baseline imbalance existed between groups, which might have introduced chance bias [37]. Such imbalances are common in trials with measurable baseline outcomes [38]. Second, statistically significant improvements were reported in salivary hemoglobin levels in the intervention group, suggesting an increase in periodontal health. The levels of salivary hemoglobin were strongly correlated with clinical measures of gingival inflammation, such as BOP. These findings are consistent with previous research showing that TPB-based education significantly reduces BOP among hospital staff [39]. Finally, although the decline in total bacterial count in the intervention group was more pronounced than in the control group, there was no significant change. This may be due to an insufficient sample size calculated based on the differences in the OHBI results. The findings of educational intervention and improved oral hygiene behavior, as well as certain oral health outcomes, emphasize the significance of behavior modification strategies and adherence to recommended oral hygiene practices.

## Limitations

This study has several limitations. First, the use of the OHBI questionnaire, which is self-reported, may have introduced social desirability effects. Participants may provide answers that do not reflect their actual behavior. This limitation was addressed by adding objective oral health outcomes as complementary measures. Second, the 3-month follow-up period may be insufficient to evaluate the effects. Third, the use of a single institution with predominance of female participants may limit the generalizability of results. Lastly, although the educational video was developed based on the TPB constructs, changes in each construct were not evaluated separately.

Future research should include longer follow-up periods to investigate the effects and maintenance of behavior change, employ more diverse populations with a balanced sex distribution to improve generalizability, and include direct evaluation of TPB constructs to provide better mechanisms of behavior change.

## Conclusion

This study demonstrated that theory-based educational intervention improved oral hygiene behaviors and led to significant improvements in certain oral health outcomes among Indonesian university students. Although baseline imbalances may have influenced some between-group comparisons, the findings highlight the potential value of incorporating psychological theory into oral health programs.

## Supplementary information

Supplementary Table 1

## Data Availability

Correspondence and requests for materials should be addressed to Daisuke Hinode.
