# Supplementary material for: Theory-based educational intervention on oral hygiene behavior among university students: a randomized controlled trial
Source: BDJ Open. 2025 Sep 26;11:80. doi: 10.1038/s41405-025-00368-y (PMC12475263; doi:10.1038/s41405-025-00368-y)
Supplement: Supplementary file 1 — Supplementary Table 1 [file 41405_2025_368_MOESM1_ESM.pdf]

Supplementary

Table 1. Demographic and oral health outcomes of the participants at baseline between completers and dropouts

Characteristics	Completers n=71	Dropouts n=9	P value
Gender, n (%)			
Female	52 (73.23)	8 (88.89)	.437[a]
Male	19 (26.76)	1 (11.11)	
Age (years), median (IQR)	20 (18, 21)	19 (19, 21)	.981[b]
Family income, n (%)			
Low	11 (15.49)	1 (11.11)	1.000[c]
Moderate	42 (59.15)	6 (66.67)	
High	18 (24.35)	2 (22.22)	
Mother education, n (%)			
Low	9 (12.68)	0 (0.00)	.780[c]
Moderate	32 (45.07)	5 (55.56)	
High	30 (42.25)	4 (44.44)	
Father education, n (%)			
Low	9 (12.68)	0 (0.00)	.780c
Moderate	32 (45.07)	5 (55.56)	
High	30 (42.25)	4 (44.44)	
Oral hygiene instruction, n (%)			
No	24 (33.80)	3 (33.33)	1.000[a]
Yes	47 (66.20)	6 (66.67)	
Dental visit, n (%)			

No	64 (90.14)	8 (88.89)	1.000[a]
Yes	7 (9.86)	1 (11.11)	
Smoking status, n (%)			
Current smoker	7 (9.86)	1 (1.11)	1.000[c]
Former smoker	5 (7.04)	0 (0.00)	
Non-smoker	59 (83.10)	8 (8.89)	
DMF-T	4 (2, 6)	3 (2, 4)	.194[b]
OHK	9 (8, 9)	9 (8, 9)	.888[b]
OHBI	10 (8, 12)	9 (8, 9)	1.000[b]
Caries activity test, n (%)			
Low	22 (30.99)	3 (33.33)	1.000[c]
Middle	34 (47.89)	4 (44.45)	
High	15 (21.12)	2 (22.22)	
Salivary haemoglobin, n (%)			
Negative	19 (26.76)	3 (3.33)	.807[c]
Positive (2μg/mL)	17 (23.94)	1 (11.11)	
Positive (5μg/mL)	35 (49.30)	5 (55.56)	
Total bacterial count (Log10 CFU/mL), median (IQR)	6.26 (5.91, 6.56)	6.44(6.16, 6.64)	.273[b]

Note. IQR, interquartile range; DMF-T, decayed, missing, and filled teeth; OHK, oral health knowledge; OHBI, oral hygiene behavior index; CFU, colony forming unit.

[a] Chi-square test

[b] Mann-Whitney test

[c] Fisher's exact test
